# Supplementary material for: Natural microbial enrichment modulates microglial states and transcriptional programs relevant to Alzheimer’s disease
Source: Front Immunol. 2026 Aug 25;17:1843174. doi: 10.3389/fimmu.2026.1843174 (PMC13549912; doi:10.3389/fimmu.2026.1843174)
Supplement: Supplementary file 1 [file DataSheet1.pdf]

frontiers

Supplementary Materials

A

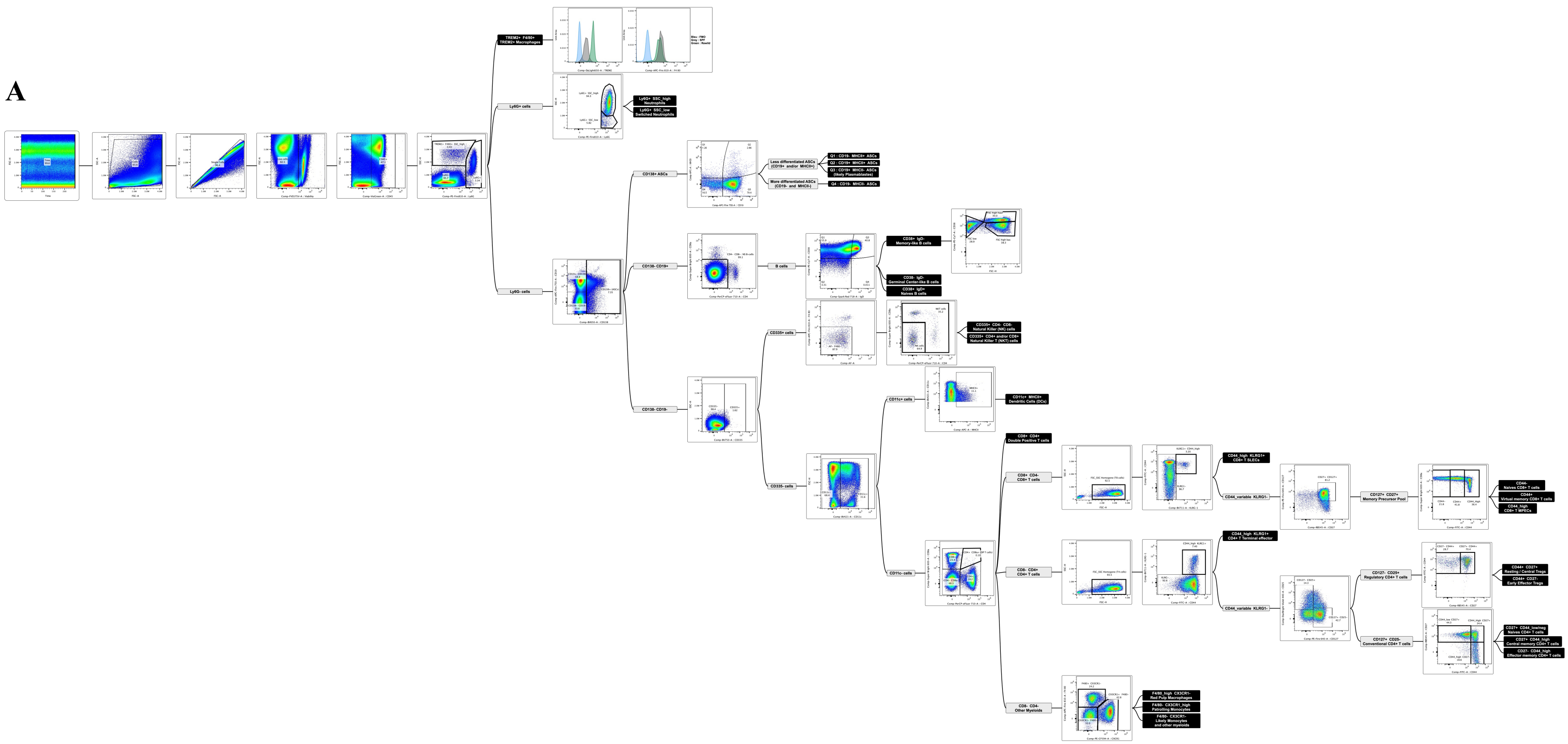

Supplementary Figure 1. Representative gating strategy for spleen and blood samples. Sequential gating was performed starting from time, FSC/SSC, singlets, live cells, and CD45$^+$ leukocytes, followed by specific lineage markers. A representative Spleen **(A)** and Blood **(B)** sample from a same Rewild-5xFAD mouse is shown. See the online version for an enlarged view.

B

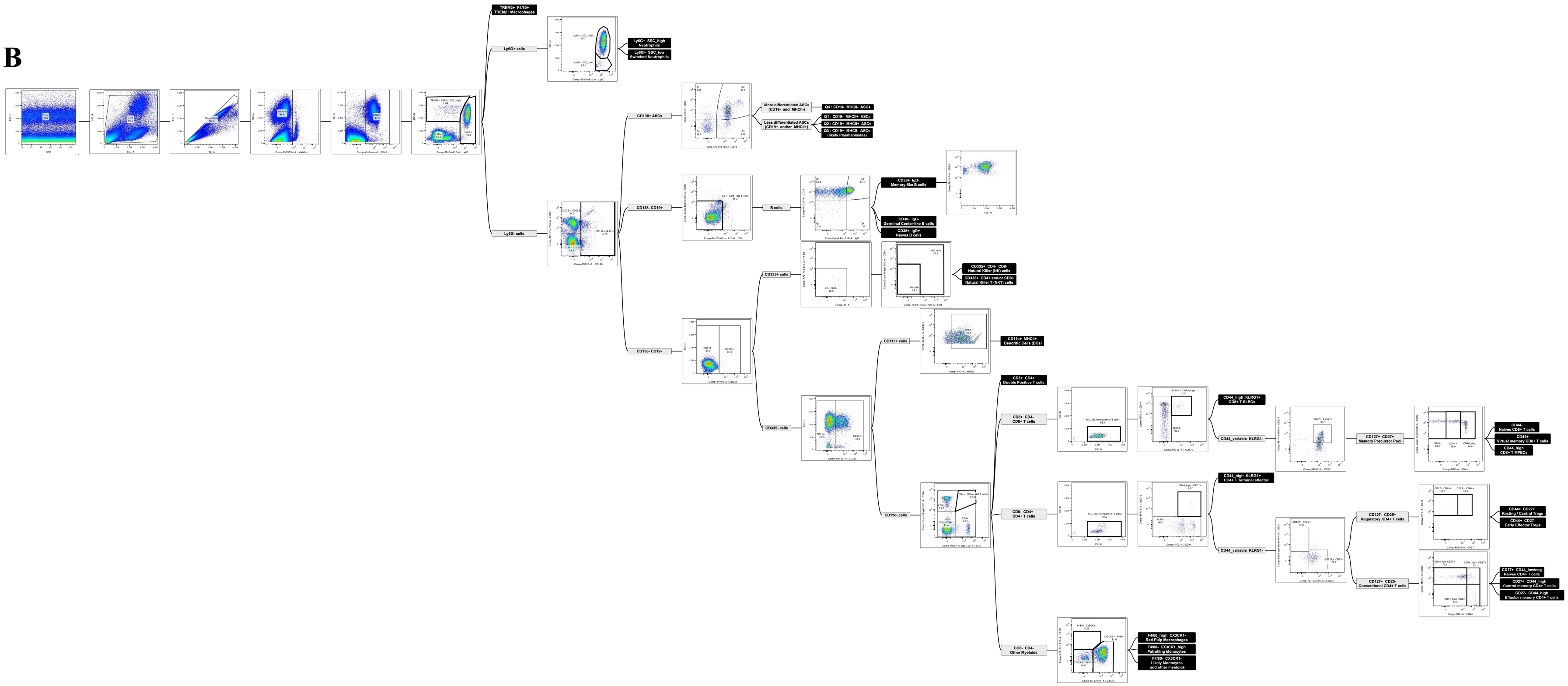

Supplementary Figure 2. Representative gating strategy for spleen and blood samples. Sequential gating was performed starting from time, FSC/SSC, singlets, live cells, and $CD45^+$ leukocytes, followed by specific lineage markers. A representative Spleen **(A)** and Blood **(B)** sample from a same Rewild-5xFAD mouse is shown. See the online version for an enlarged view.

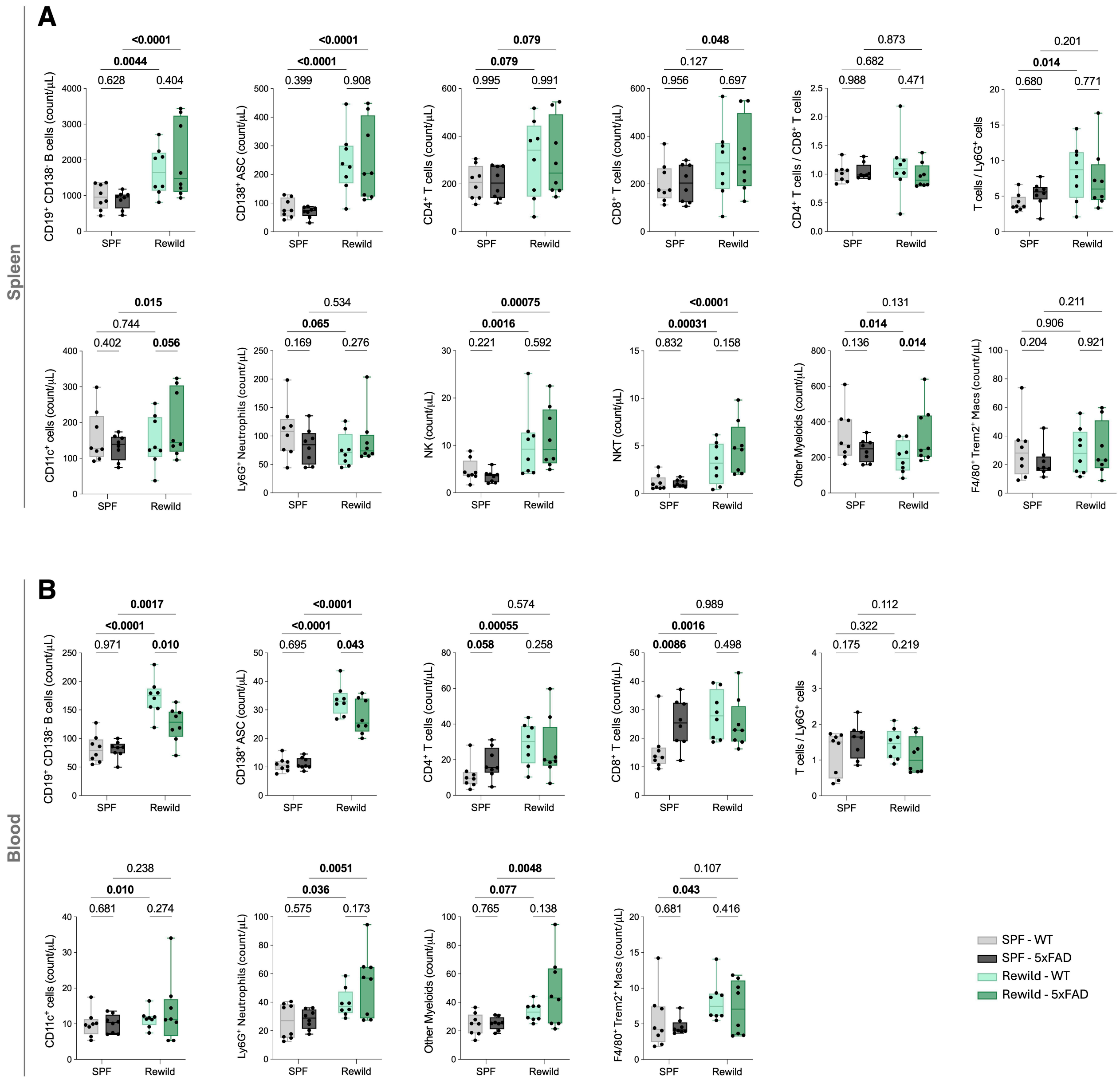

Supplementary Figure 2. Counts of leukocyte subsets in spleen and blood. Flow cytometry-based cell number (count per µL) of major leukocyte populations measured in spleen **(A)** and blood **(B)** from female mice housed under SPF conditions or in the indoor vivarium (Rewild): SPF-WT, SPF-5xFAD, Rewild-WT, and Rewild-5xFAD (N = 8 per group). The total T-cell/Ly6G$^+$ cell ratio and the CD4+/CD8+ T-cell ratio are included where indicated. Statistical analyses were performed as described in Methods. Ratios were analyzed after log10 transformation, whereas raw ratio values are displayed.

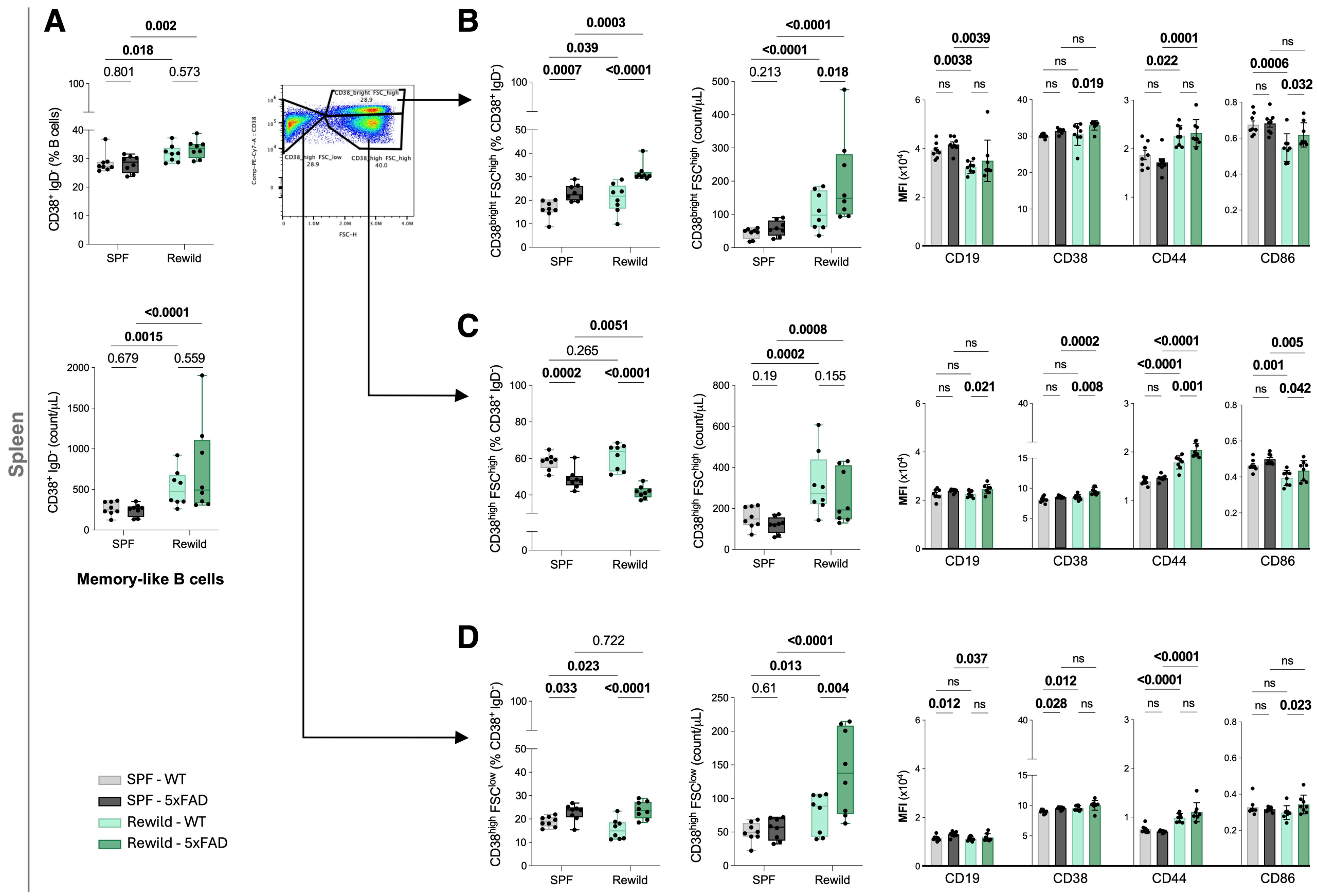

Supplementary Figure 3. CD38/FSC-defined heterogeneity within splenic memory-like B cells. Flow cytometry analysis of splenic memory-like B cells from female SPF-WT, SPF-5xFAD, Rewild-WT, and Rewild-5xFAD mice (N = 8 per group). Memory-like B cells were operationally defined as $CD38^{+}IgD^{-}$ and were further partitioned based on CD38 intensity and cell size (FSC-H). **(A)** Total $CD38^{+}IgD^{-}$ memory-like B cells in spleen shown as cell numbers (count per µL) and as proportions of B lymphocytes, with representative gating illustrating the CD38/FSC-based subdivision quantified in panels B-D. **(B-D)** Quantification of CD38/FSC-defined subfractions within the memory-like compartment: $CD38^{bright}FSC^{high}$ **(B)**, $CD38^{high}FSC^{high}$ **(C)**, and $CD38^{high}FSC^{low}$ **(D)**. For each subfraction, results are shown as numbers (counts/µL) and as proportions of $CD38^{+}IgD^{-}$ cells, alongside marker expression (MFI) for CD19, CD38, CD44, and CD86. Statistical analyses were performed as described in Methods.

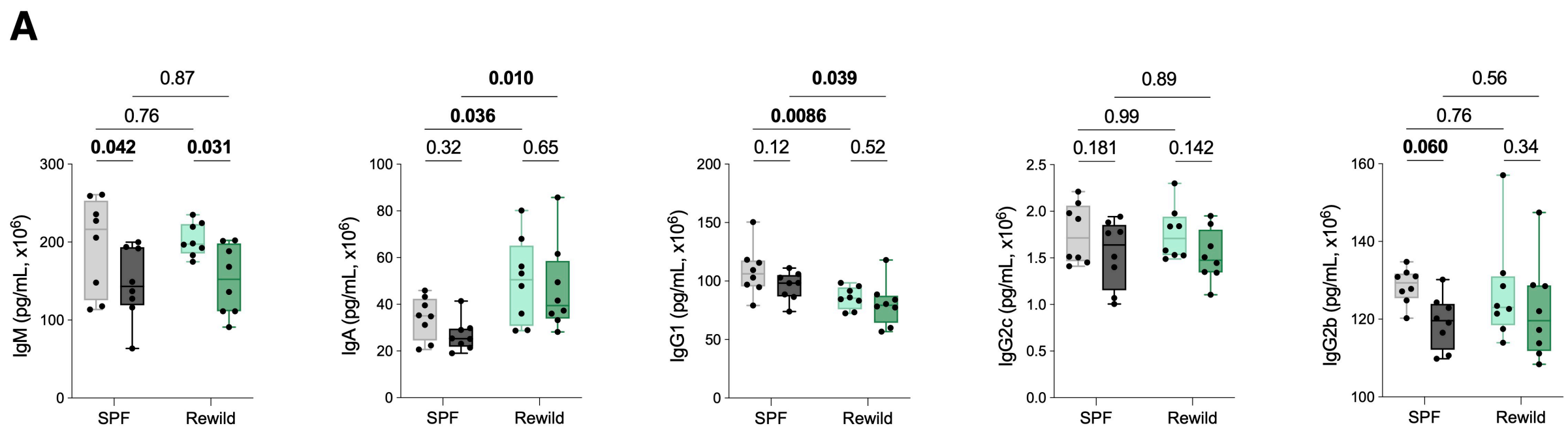

Supplementary Figure 4. Plasma immunoglobulins profiling. Plasma immunoglobulin concentrations (IgM, IgA, IgG1, IgG2b, IgG2c) were quantified by Meso Scale Discovery (MSD) immunoassays in female SPF-WT, SPF-5xFAD, Rewild-WT, and Rewild-5xFAD mice (N = 8; flow cytometry cohort). Values are reported as pg/mL (x10^6) as indicated. Statistical testing was performed on $\log_{10}$-transformed data using a two-way ANOVA.

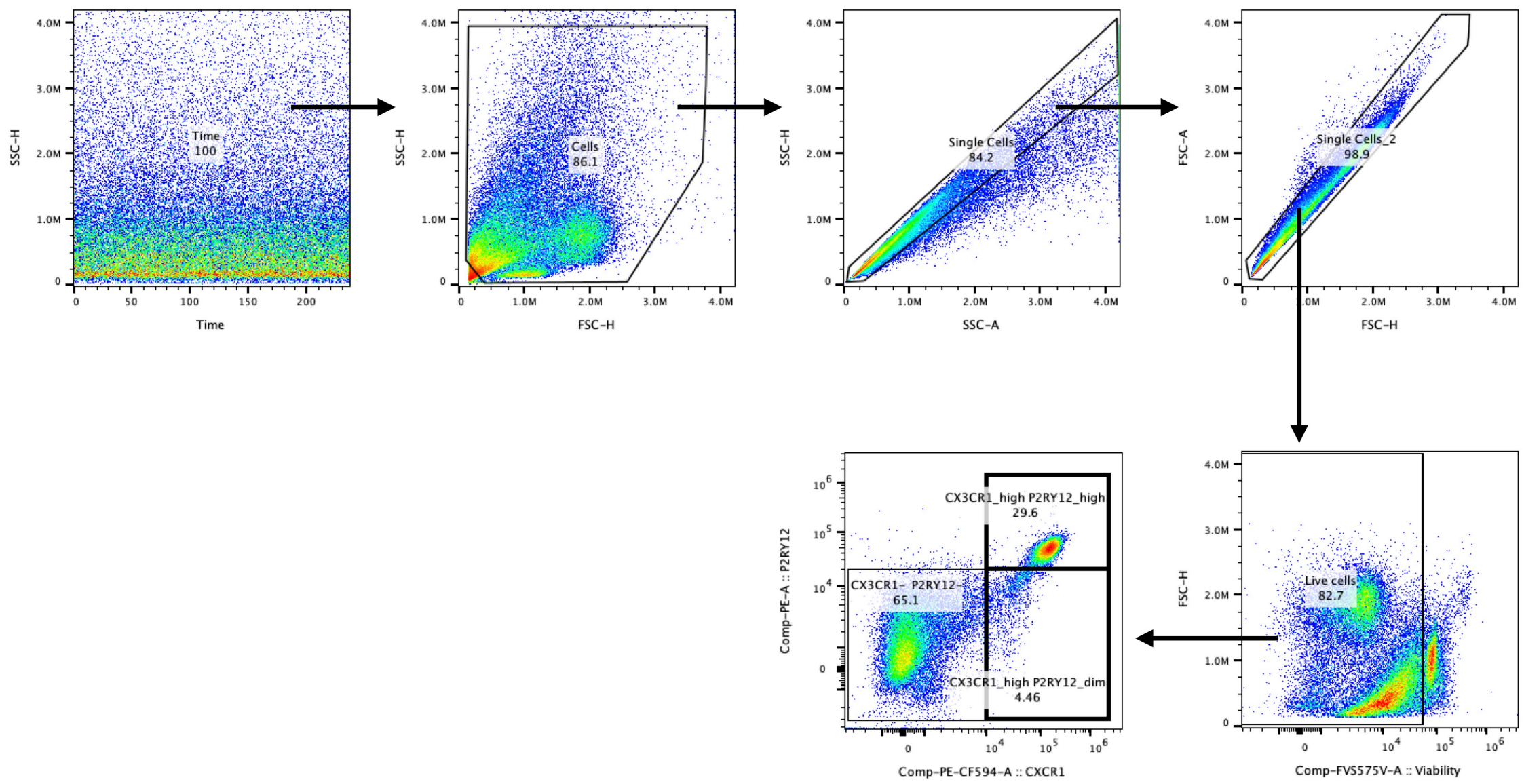

Supplementary Figure 5. Representative gating strategy for brain samples. Sequential gating was performed starting from time, FSC/SSC, singlets, live cells, then identification of microglia via P2RY12 vs CX3CR1 plot.

Supplementary Table 1. List of fluorochrome-conjugated antibodies and reagents used in flow cytometry for blood, spleen and brain leukocyte profiling.

Antigen / Reagent	Fluorochrome	Supplier	# Catalog
**Fixable Viability Stain**	FVS575V	BD Biosciences	565694
**TREM2**	DyLight 650	Novus	FAB17291W
**P2RY12**	PE	BioLegend	848004
**MHCII**	APC	Invitrogen	APC-65122-100UG
**Ly6g**	PE-Fire 810	BioLegend	127673
**KLRG1**	Brilliant Violet 711	BD Biosciences	564014
**IgD**	AlexaFluor700	BioLegend	405730
**Fc block**	-	BioLegend	103116
**F4/80**	APC-Fire 810	BioLegend	123166
**CX3CR1**	PE-CF 594	BD Biosciences	567823
**CD8a**	Super Bright 600	Invitrogen	63-0081-82
**CD86**	VioBright 515	Miltenyi Biotec	130-122-136
**CD45**	VioGreen	Miltenyi Biotec	130-123-900
**CD44**	FITC	Invitrogen	11-0441-82
**CD4**	PerCP-eFluor 710	Invitrogen	46-0041-82
**CD38**	BrilliantViolet 480	Invitrogen	414-0381-82
**CD335**	Brilliant Violet 750	BD Biosciences	746875
**CD27**	RB545	BD Biosciences	756643
**CD25**	StarBright Violet 440	Bio-Rad	MCA1260SBV440
**CD19**	APC-Fire 750	BioLegend	115558
**CD138**	Brilliant Violet 650	BioLegend	142518
**CD127**	PE-Fire 640	BioLegend	158214
**CD11c**	BV421	Invitrogen	404-0114-82
**CD11b**	StarBright Blue 700	Bio-Rad	MCA711SBB700

Supplementary Table 2. List of forward and reverse primers designed for amplification of murine cytokine, chemokine and housekeeping genes. Primer sequences were used to quantify mRNA expression in brain tissue by SYBR Green-based qRT-PCR.

	Forward	Reverse
**Target murine genes**		
**Tnf**	TGCCTATGTCTCAGCCTCTTC	GAGGCCATTTGGGAACTTCT
**Clec7a**	TGCCTATGTCTCAGCCTCTTC	GAGGCCATTTGGGAACTTCT
**IL-1a**	ATGGTTCTGGGAGGATGGAT	GCTTTCCTGGGGAGCTGTAT
**IL-1b**	TTGGTTAAATGACCTGCAACA	GAGCGCTCACGAACAGTTG
**Nlrp3**	ATCTTTTGGGGTCCGTCAACT	GCAACTGTTCCTGAACTCAACT
**IL-6**	ATTACCCGCCCGAGAAAGG	CATGAGTGTGGCTAGATCCAAG
**Ccl2**	ACCACGGCCTTCCCTACTTC	TCCACGATTTCCCAGAGAACA
**Itgax**	GCTACAAGAGGATCACCAGCAG	GTCTGGACCCATTCCTTCTTGG
**IL-10**	ATGGAGCCTCAAGACAGGAC	GGATCTGGGATGCTGAAATC
**Housekeeping murine gene**		
**Gapdh**	ACAAAATGGTGAAGGTCGGTG	TGGCAACAATCTCCACTTTGC

Supplementary Table 3. List of primary and secondary antibodies used for protein detection by immunoblot. Dilutions used, host species, suppliers, and catalog numbers are indicated.

Protein	Dilution	Species	Supplier	# Catalog
**Primary antibodies**				
**Iba1**	1/1000	Mouse monoclonal	Invitrogen	MA5-27726
**Gfap**	1/1000	Mouse monoclonal	BioLegends	835301
**Secondary antibodies**				
**HRP-conjugated IgG (HC+LC)**	1/5000	Goat anti-mouse	Jackson laboratories	115-035-003
